# Volumosa Calcificação Caseosa do Anel Mitral em Localização Atípica

**DOI:** 10.36660/abc.20210949

**Published:** 2022-11-23

**Authors:** Joana Laranjeira Correia, Miguel Correia

**Affiliations:** 1 Centro Hospitalar Tondela Viseu Portugal Centro Hospitalar Tondela –Viseu, Viseu – Portugal; 2 Hospital Cuf Viseu Viseu Portugal Hospital Cuf Viseu, Viseu – Portugal

**Keywords:** Técnicas de Imagem Cardíaca, Tomografia Computadorizada por Raios X, Acidente Vascular Cerebral

## Abstract

A calcificação do anel mitral (CAM) é um processo fibrótico crônico e degenerativo comumente observado da base da valva mitral, geralmente considerado um achado incidental. Embora inicialmente a CAM fosse considerada uma consequência de um processo degenerativo relacionado à idade, achados recentes sugerem outros mecanismos contributivos independentes, como aterosclerose e metabolismo anormal de cálcio-fósforo.

A calcificação caseosa do anel mitral (cCAM) é uma variante raramente descrita da CAM, caracterizada por uma massa ovoide, focal, com calcificações internas semelhantes a líquido caseoso e detritos.

Diferenciar um cCAM de outras massas cardíacas aderidas ao anel mitral pode ser um desafio. Uma única modalidade de imagem, como o ecocardiograma transtorácico, pode não ser suficiente para um diagnóstico claro. Portanto, uma abordagem de imagem multimodal é necessária, incluindo tomografia computadorizada cardíaca e ressonância magnética cardíaca (RMC).

A CAM e a cCAM afetam tipicamente o anel mitral posterior, com poucos casos na literatura descrevendo o envolvimento do anel anterior. Apresentamos um caso raro de calcificação caseosa do anel mitral anterior encontrado em uma RMC realizada para avaliar uma massa atrial esquerda identificada em um ecocardiograma transtorácico.

## Caso clínico

Uma mulher de 84 anos, com história médica conhecida de hipertensão e dislipidemia, foi encaminhada à nossa unidade para realização de ressonância magnética cardiovascular (RMC) para posterior avaliação de massa em átrio esquerdo evidenciada por ecocardiograma transtorácico, realizado no contexto de hospitalização por acidente vascular cerebral isquêmico.

**Figura 1:**
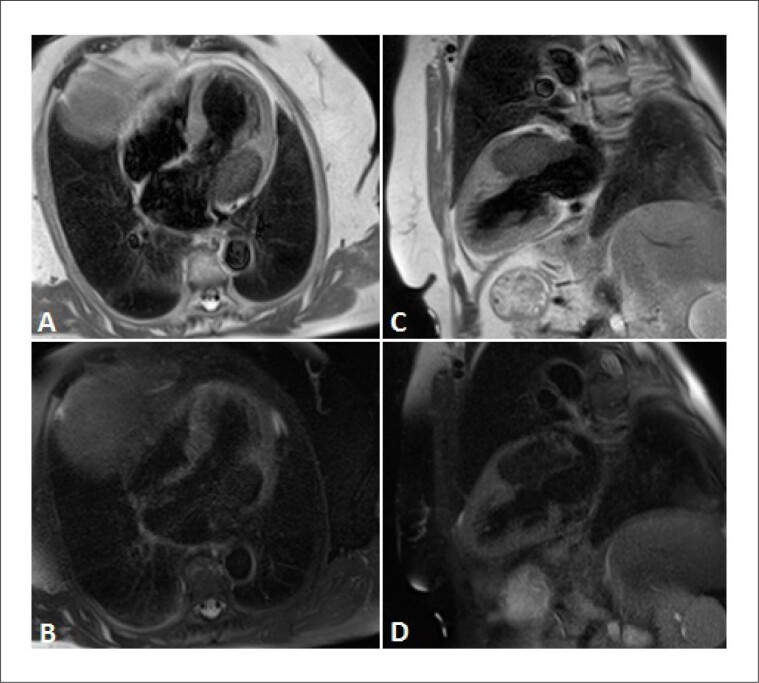
Imagens Fast Spin Echo (FSE) em cortes de eixo longo de 2 e 4 câmeras. (A, B): T1 FSE mostrando uma massa hipointensa em relação ao miocárdio (seta). (C, D): T2 FSE com pulso de saturação de gordura mostrando quase ausência de sinal no local da massa.

**Figura 2:**
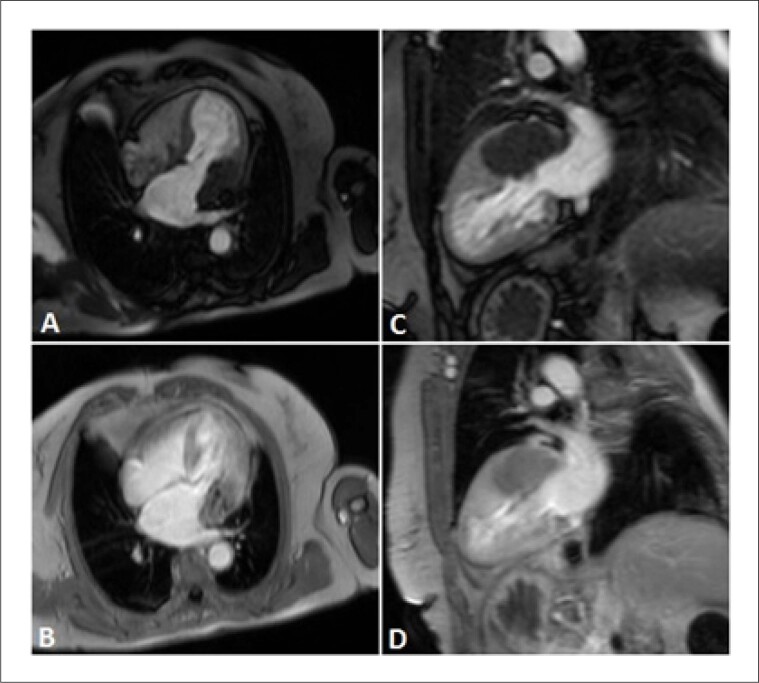
Imagens de perfusão de primeira passagem (A, C) e sequência de realce precoce (B, D) nas incidências de eixo longo de 2 e 4 câmeras, demonstrando ausência de perfusão ou acúmulo de contraste na massa.

**Figura 3:**
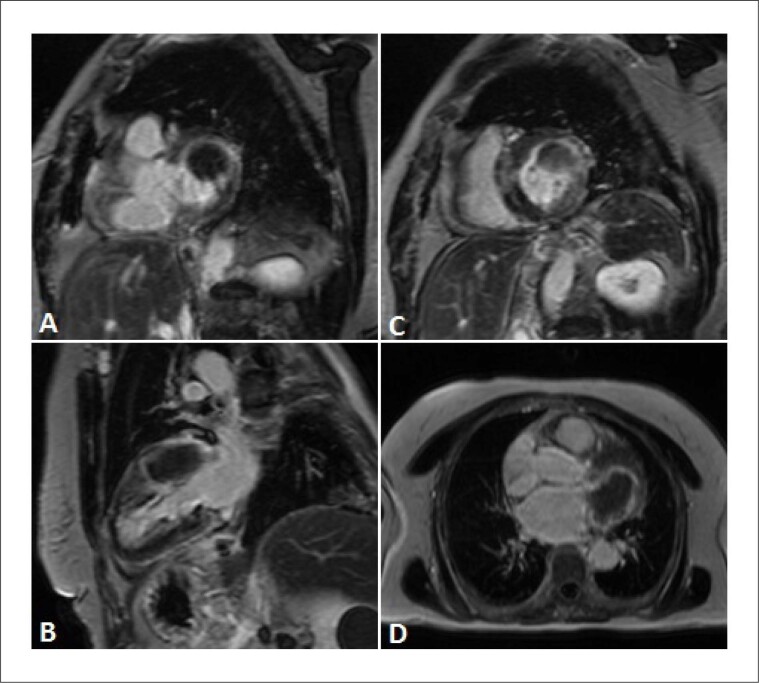
Padrões de realce tardio em diferentes planos: (A, C) - eixo curto; B - eixo longo de 2 câmaras; D - corte basal transaxial. Todos mostram apenas uma pequena borda de realce na periferia da massa.

A RMC revelou uma massa muito grande no sulco auriculoventricular ântero-lateral esquerdo, hipointensa em todas as sequências rápidas spin-eco (Figura 1) e não exibiu perfusão ou evidência de captação de contraste nas sequências de realce precoce (Figura 2). A massa era ligeiramente mais escura que o miocárdio nas sequências cine (precessão livre em estado estacionário). As sequências de realce tardio (Figura 3) mostraram uma pequena borda aprimorada em torno de um importante núcleo não aprimorado.

**Figura 4:**
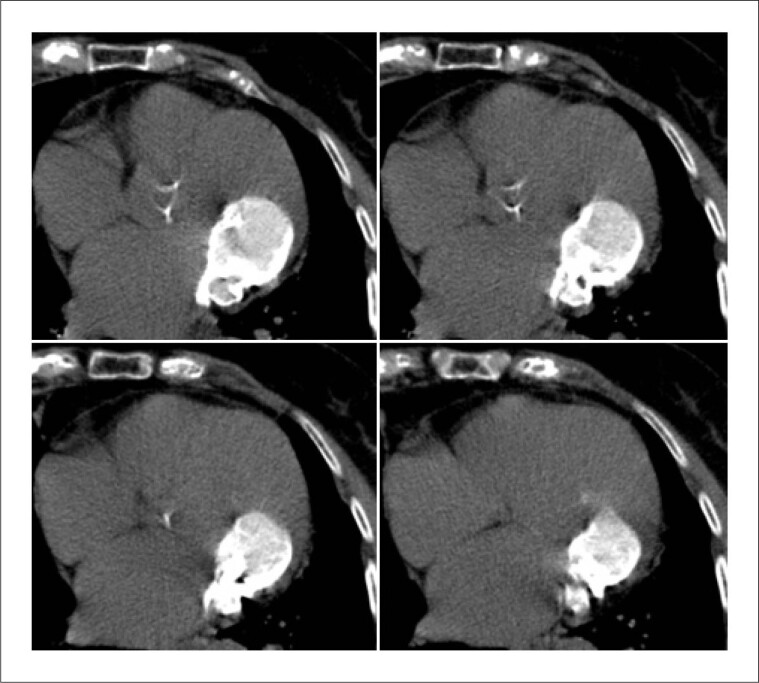
Imagens de TC cardíacas em planos axiais contíguos, mostrando grande massa calcificada com baixa atenuação central.

Com base na literatura existente, apesar da localização atípica, as características da massa na RMC poderiam ser compatíveis com calcificação caseosa do anel mitral (cCAM).^1–3^ Para confirmar essa hipótese, o paciente foi então submetido a uma tomografia computadorizada cardíaca adicional (TC) (aquisição prospectiva sem contraste, cobrindo todo o coração), que evidenciou massa calcificada com menor atenuação na parte central (Figura 4), características tipicamente encontradas na calcificação caseosa do anel mitral anterior.

## Discussão

A calcificação do anel mitral (CAM) é um processo fibrótico crônico e degenerativo comumente observado da base da valva mitral, geralmente considerado um achado incidental.^4^ A CAM é mais prevalente em idosos, principalmente em mulheres.^5^ A prevalência de CAM varia de 5% a 42%, dependendo da modalidade de imagem utilizada.^6^

A CAM é definida como o acúmulo de cálcio ao longo do anel. Embora inicialmente a CAM fosse considerada uma consequência de um processo degenerativo relacionado à idade, achados recentes sugerem outros mecanismos contributivos independentes, como aterosclerose e metabolismo anormal de cálcio-fósforo.^4,6^ A CAM pode ocorrer em pacientes mais jovens com doença renal avançada ou outros distúrbios metabólicos que resultam em metabolismo anormal do cálcio.^5^ Os fatores contribuintes incluem fatores relacionados à idade, fatores de risco cardiovascular, aumento do estresse da válvula mitral (hipertensão, estenose aórtica e cardiomiopatia hipertrófica), metabolismo anormal de cálcio-fósforo, distúrbios congênitos (síndrome de Marfan, síndrome de Hurler) e sexo feminino.^4^

O prognóstico do CAM relaciona-se com a associação com eventos adversos cardiovasculares e mortalidade e a disfunção valvar mitral que pode causar. A CAM tem sido independentemente associada à mortalidade por todas as causas e cardiovascular, com um risco aumentado de eventos de doença cardíaca coronária e insuficiência cardíaca incidente. Além disso, a associação com AVC ocorre em múltiplas coortes, parcialmente relacionada ao risco de fibrilação atrial por disfunção valvar progressiva. A CAM também está associada a um aumento da prevalência de atrasos do sistema de condução, incluindo bloqueio atrioventricular, bloqueio de ramo e atraso de condução intraventricular. A CAM geralmente tem pouco ou nenhum impacto na hemodinâmica do influxo ventricular esquerdo ou na função da válvula mitral. Dados limitados sugerem que a CAM pode exacerbar a regurgitação mitral, e raramente foi relatada associação com estenose mitral. Por fim, a CAM pode ser um fator importante no desenvolvimento da endocardite da válvula mitral, atuando como um nicho para infecção.^4,6^

A cCAM é uma variante raramente descrita de CAM, caracterizada por uma massa ovoide, focal, com calcificações internas semelhantes a líquido caseoso e detritos.^1^ A terminologia de cCAM é peculiar, pois o termo caseoso geralmente se refere a um tipo de necrose frequentemente encontrada na tuberculose. A apresentação clínica mais comum é o achado incidental de uma massa intracardíaca durante a imagem cardíaca.^5^ A prevalência ecocardiográfica é de 0,6% em pacientes com CAM, e a prevalência geral é de até 0,07% na população geral.^7^ A cCAM tende a ocorrer em pacientes idosos e está associada à hipertensão.^8^

A cCAM pode mimetizar massas cardíacas como tumores (mais comumente mixoma), abscessos e vegetações. Diferenciar uma cCAM de outras massas cardíacas aderidas ao anel mitral pode ser um desafio devido às suas características de imagem variáveis, dependendo do seu estágio de evolução. Uma única modalidade de imagem, como a ecocardiografia, muitas vezes é insuficiente para um diagnóstico correto. Portanto, a abordagem de imagem multimodal é obrigatória.^4,5^

Devido ao elevado teor de cálcio, a CAM é geralmente hipointensa na RMC. No entanto, os sais de cálcio e o fluido proteináceo na CAM caseosa podem gerar um sinal alto nas sequências spin-eco ponderadas em T1 (Tabela I).^1,2^

**Tabela 1:** Aspectos da ressonância magnética cardíaca encontrados nos casos relatados de calcificação caseosa do anel mitral

Pré-contraste ponderado em T1	Pré-contraste ponderado em T2	BSSFP pré-contraste	Perfusão de primeira passagem	Melhoria atrasada
Escuro	Preto	Ligeiramente mais escuro que o miocárdio	Não perfundido	Borda aprimorada em torno de um núcleo não aprimorado

BSSFP: precessão livre em estado estacionário equilibrado. Adaptado de Monti el al.^2^

A CAM e a cCAM comumente afetam o anel mitral posterior, com poucos casos na literatura descrevendo o envolvimento do anel anterior.^3,7^ A cCAM pode levar à doença valvar mitral (regurgitação ou estenose) ou embolização sistêmica. Neste caso clínico, a condição que motivou a abordagem diagnóstica foi o acidente vascular cerebral. Os mecanismos postulados de embolização incluem embolização de pequenas partes calcificadas, ulceração da superfície complicada pela formação de trombos e subsequente embolização ou fistulização da necrose caseosa no lúmen do átrio esquerdo ou ventrículo esquerdo.^5,9^ A maioria dos autores concorda que o tratamento cirúrgico é indicado em pacientes sintomáticos com cCMA associada à disfunção valvar mitral, manifestações embólicas ou quando é impossível descartar a possibilidade de um tumor.^9^

Em conclusão, a CAM é uma desordem incompletamente compreendida com implicações clínicas e prognósticas. Embora a presença de cCAM possa representar um dilema diagnóstico, a compreensão dessa entidade permite estabelecer um diagnóstico preciso.^1^

## References

[1] Shriki J, Rongey C, Ghosh B, Daneshvar S, Colletti PM, Farvid A (2010). Caseous Mitral Annular Calcifications: Multimodality Imaging Characteristics. World J Radiol.

[2] Monti L, Renifilo E, Profili M, Balzarini L (2008). Cardiovascular Magnetic Resonance Features of Caseous Calcification of the Mitral Annulus. J Cardiovasc Magn Reson.

[3] Lee C, Yoon AJ, Klipfel NE, Cunningham MJ, Saremi F (2012). Caseous Mitral Annular Calcification Along the Anterolateral Annulus Causing Mild Mitral Regurgitation: Multi-Modality Imaging and Diagnosis. Circ J.

[4] Abramowitz Y, Jilaihawi H, Chakravarty T, Mack MJ, Makkar RR (2015). Mitral Annulus Calcification. J Am Coll Cardiol.

[5] Elgendy IY, Conti CR (2013). Caseous Calcification of the Mitral Annulus: A Review. Clin Cardiol.

[6] Massera D, Kizer JR, Dweck MR (2020). Mechanisms of Mitral Annular Calcification. Trends Cardiovasc Med.

[7] Stamou SC, Braverman AC, Kouchoukos NT (2010). Caseous Calcification of the Anterior Mitral Valve Annulus Presenting as Intracardiac Mass. J Thorac Cardiovasc Surg.

[8] Deluca G, Correale M, Ieva R, Del Salvatore B, Gramenzi S, Di Biase M (2008). The Incidence and Clinical Course of Caseous Calcification of the Mitral Annulus: A Prospective Echocardiographic Study. J Am Soc Echocardiogr.

[9] Dietl CA, Hawthorn CM, Raizada V (2016). Risk of Cerebral Embolization with Caseous Calcification of the Mitral Annulus: Review Article. Open Cardiovasc Med J.

