# Supplementary material for: Volumosa Calcificação Caseosa do Anel Mitral em Localização Atípica
Source: Arq Bras Cardiol. 2022 Nov 23;119(6):1002–5. [Article in Portuguese] doi: 10.36660/abc.20210949 (PMC9814819; doi:10.36660/abc.20210949)
Supplement: Translation

# Translation: Bulky Mitral Annulus Caseous Calcification in an Atypical Location

**Authors:** Joana Laranjeira Correia, Miguel Correia

**Affiliations:** 1 Viseu Portugal Centro Hospitalar Tondela – Viseu, Viseu – Portugal; 2 Viseu Portugal Hospital Cuf Viseu, Viseu – Portugal

**Keywords:** Cardiac Imaging Techniques, Tomography, X-Ray Computed, Stroke

## Abstract

Mitral annular calcification (MAC) is a commonly observed chronic and degenerative fibrotic process of the base of the mitral valve, usually deemed as an incidental finding. Although initially, MAC was thought to be a consequence of an age-related degenerative process, recent findings suggest other independent contributive mechanisms, such as atherosclerosis and abnormal calcium-phosphorus metabolism.

Caseous calcification of the mitral annulus (cMAC) is a rarely described variant of MAC, characterized by an ovoid, focal mass with internal caseous fluid-like calcifications and debris.

Differentiating a cMAC from other cardiac masses attached to the mitral annulus may be challenging. A single imaging modality, such as transthoracic echocardiography, may not be sufficient for a clear diagnosis. Therefore, a multimodal imaging approach is necessary, including cardiac tomography computerized imaging and cardiac magnetic resonance (CMR).

MAC and cMAC typically affect the posterior mitral annulus, with very few cases in the literature describing the involvement of the anterior annulus. We present a rare case of an anterior mitral annulus caseous calcification found in a CMR performed to evaluate a left atrial mass identified on a transthoracic echocardiogram.

## Clinical case

An 84-year-old woman with a known medical history of hypertension and dyslipidemia was referred to our unit to perform a cardiovascular magnetic resonance (CMR) for further evaluation of a left atrial mass depicted by transthoracic echocardiography, which was performed in the context of hospitalization by an ischemic stroke.

CMR revealed a very large mass on the left anterolateral auriculoventricular groove that was hypointense in all fast spin-echo sequences (Figure 1) and exhibited no perfusion or evidence of contrast uptake in early enhancement sequences (Figure 2). The mass was slightly darker than the myocardium in the cine sequences (steady-state free precession). Delayed enhancement sequences (Figure 3) showed a small, enhanced border surrounding an important non-enhanced core.

**Figure 1:** Fast spin echo (FSE) images in 2 and 4 chamber long axis views. (A, B): T1 FSE showing a hypointense mass relative to the myocardium (arrow). (C,D): T2 FSE with fat saturation pulse showing almost absence of signal in the mass location.

**Figure 2:** First pass perfusion (A, C) and early enhancement sequence (B, D) images in 2 chamber and 4 chamber long axis views, demonstrating absence of perfusion or contrast accumulation in the mass.

**Figure 3:** Delayed enhancement patterns in different planes: (A, C) - short axis; B - 2 chamber long axis; D - transaxial basal slice. All show only a small rim of enhancement at the mass periphery.

Based on the existing literature, although the atypical location, the mass characteristics on CMR could be compatible with caseous calcification of the mitral annulus (cMAC).^1–3^ To confirm this hypothesis, the patient was then submitted to an additional cardiac computerized tomography (CT) scan (prospective acquisition without contrast, covering all heart), that showed a calcified mass with less attenuation in the central part (Figure 4), features typically found on caseous calcification of the anterior mitral annulus.

**Figure 4:** Cardiac CT images in contiguous axial planes, showing a large calcified mass with low central attenuation.

## Discussion

Mitral annular calcification (MAC) is a commonly observed chronic and degenerative fibrotic process of the base of the mitral valve, usually deemed as an incidental finding.^4^ MAC is more prevalent in the elderly, particularly in women.^5^ The prevalence of MAC varies from 5% to 42%, depending on the imaging modality used.^6^

MAC is defined as the accumulation of calcium along the annulus. Although initially, MAC was thought to be a consequence of an age-related degenerative process, recent findings suggest other independent contributive mechanisms, such as atherosclerosis and abnormal calcium-phosphorus metabolism.^4,6^ MAC may occur in younger patients with advanced renal disease or other metabolic disorders that result in abnormal calcium metabolism.^5^ The contributing factors include age-related factors, cardiovascular risk factors, increased mitral valve stress (hypertension, aortic stenosis, and hypertrophic cardiomyopathy), abnormal calcium-phosphorus metabolism, congenital disorders (Marfan syndrome, Hurler syndrome) and female sex.^4^

The prognosis of MAC relates to the association with cardiovascular adverse events and mortality and the mitral valve dysfunction it can cause. MAC has been independently associated with all-cause and cardiovascular mortality, with an increased risk of coronary heart disease events and incident heart failure. Moreover, the association with stroke occurs in multiple cohorts, partially related to the risk of atrial fibrillation due to progressive valvular dysfunction. MAC is also associated with an increased prevalence of conduction system delays, including atrioventricular block, bundle branch block, and intraventricular conduction delay. MAC generally has little or no impact on left ventricular inflow hemodynamics or mitral valve function. Limited data suggest that MAC may exacerbate mitral regurgitation, and rarely association with mitral stenosis has been reported. Lastly, MAC may be an important factor in the development of mitral valve endocarditis by acting as a nidus for infection.^4,6^

cMAC is a rarely described variant of MAC, characterized by an ovoid, focal mass with internal caseous fluid-like calcifications and debris.^1^ The terminology of cMAC is peculiar, as the term caseous usually refers to a type of necrosis often encountered in tuberculosis. The most common clinical presentation is the incidental finding of an intracardiac mass during cardiac imaging.^5^ The echocardiographic prevalence is 0.6% in patients with MAC, and the overall prevalence is up to 0.07% in the general population.^7^ cMAC tends to occur in older patients and is associated with hypertension.^8^

cMAC may mimic cardiac masses such as tumors (most commonly myxoma), abscesses and vegetations. Differentiating a cMAC from other cardiac masses attached to the mitral annulus may be challenging due to its variable imaging characteristics depending on its stage of evolution. A single imaging modality, such as echocardiography, is often insufficient for a correct diagnosis. Therefore, the multimodal image approach is mandatory.^4,5^

Due to the elevated calcium content, MAC is generally hypointense on CMR. Nevertheless, the calcium salts and proteinaceous fluid in caseous MAC can generate a high signal on T1 weighted spin-echo sequences (Table I).^1,2^

**Table 1:** Cardiac magnetic resonance appearances found in the reported cases of caseous calcification of the mitral annulus

Pre-contrast T1-weighted	Pre-contrast T2-weighted	Pre-contrast BSSFP	First pass perfusion	Delayed Enhancement
Dark	Black	Slightly darker than myocardium	Not perfused	Enhanced border surrounding a non-enhanced core

BSSFP: balanced steady state free precession. Adapted from from Monti et al.^2^

MAC and cMAC commonly affect the posterior mitral annulus, with very few cases in the literature describing the involvement of the anterior annulus.^3,7^ cMAC can lead to mitral valve disease (regurgitation or stenosis) or systemic embolization. In this clinical case, the condition that motivated the diagnostic approach was a stroke. The postulated mechanisms of embolization include embolization of small calcified parts, ulceration of the surface complicated by thrombus formation, and subsequent embolization or fistulization of the caseous necrosis in the lumen of the left atrium or left ventricle.^5,9^ Most authors agree that surgical treatment is indicated in symptomatic patients with cCMA associated with mitral valvular dysfunction, embolic manifestations, or when it is impossible to rule out the possibility of a tumor.^9^

In conclusion, MAC is an incompletely understood disorder with clinical and prognostic implications. Although the presence of cMAC may pose a diagnostic dilemma, understanding this entity allows for establishing an accurate diagnosis.^1^
